# Acoustic-based machine learning approaches for depression detection in Chinese university students

**DOI:** 10.3389/fpubh.2025.1561332

**Published:** 2025-05-15

**Authors:** Yange Wei, Shisen Qin, Fengyi Liu, Rongxun Liu, Yunze Zhou, Yuanle Chen, Xingliang Xiong, Wei Zheng, Guangjun Ji, Yong Meng, Fei Wang, Ruiling Zhang

**Affiliations:** ^1^Department of Early Intervention, Mental Health and Artificial Intelligence Research Center, The Second Affiliated Hospital of Xinxiang Medical University, Henan Mental Hospital, Xinxiang, China; ^2^Peking University Sixth Hospital, Peking University Institute of Mental Health, NHC Key Laboratory of Mental Health (Peking University), National Clinical Research Center for Mental Disorders (Peking University Sixth Hospital), Beijing, China; ^3^School of Public Health, Xinxiang Medical University, Xinxiang, China; ^4^School of Psychology, Xinxiang Medical University, Xinxiang, China; ^5^Department of Psychiatry, The Affiliated Brain Hospital, Guangzhou Medical University, Guangzhou, China; ^6^Department of Early Intervention, Nanjing Brain Hospital, Nanjing Medical University, Nanjing, China

**Keywords:** depression, acoustic features, machine learning, Chinese university students, campuses

## Abstract

**Background:**

Depression is major global public health problems among university students. Currently, the evaluation and monitoring of depression predominantly depend on subjective and self-reported methods. There is an urgent necessity to develop objective means of identifying depression. Acoustic features, which convey emotional information, have the potential to enhance the objectivity of depression assessments. This study aimed to investigate the feasibility of utilizing acoustic features for the objective and automated identification and characterization of depression among Chinese university students.

**Methods:**

A cross-sectional study was undertaken involving 103 students with depression and 103 controls matched for age, gender, and education. Participants' voices were recorded using a smartphone as they read neutral texts. Acoustic analysis and feature extraction were performed using the OpenSMILE toolkit, yielding 523 features encompassing spectral, glottal, and prosodic characteristics. These extracted acoustic features were utilized for discriminant analysis between depression and control groups. Pearson correlation analyses were conducted to evaluate the relationship between acoustic features and Patient Health Questionnaire-9 (PHQ-9) scores. Five machine learning algorithms including Linear Discriminant Analysis (LDA), Logistic Regression, Support Vector Classification, Naive Bayes, and Random Forest were used to perform the classification. For training and testing, ten-fold cross-validation was employed. Model performance was assessed using receiver operating characteristic (ROC) curve, area under the curve (AUC), precision, accuracy, recall, and F1 score. Shapley Additive exPlanations (SHAP) method was used for model interpretation.

**Results:**

In depression group, 32 acoustic features (25 spectral features, 5 prosodic features and 2 glottal features) showed significant alterations compared with controls. Further, 27 acoustic features (10 spectral features, 3 prosodic features, and 1 glottal features) were significantly correlated with depression severity. Among five machine learning algorithms, LDA model demonstrated the highest classification performance, with an AUC of 0.771. SHAP analysis suggested that Mel-frequency cepstral coefficients (MFCC) features contributed most to the model's classification efficacy.

**Conclusions:**

The integration of acoustic features and LDA model demonstrates a high accuracy in distinguishing depression among Chinese university students, suggesting its potential utility in rapid and large-scale depression screening. MFCC may serve as objective and valid features for the automated identification of depression on Chinese university campuses.

## 1 Introduction

The rising prevalence of depression among Chinese university students underscores the critical necessity for an effective system to identify depression. The university period is a crucial stage in the transition from student identity to social identity, students face heavy academic workloads, diverse course offerings, and intense competition (1, 2). The prevalence of depression among Chinese university students exceeds 20% (3, 4), with 11% exhibiting suicidal ideation (5). Depression in university students typically manifests as loss of pleasure, interest, energy, and appetite, reduced attention and concentration, and insomnia (6, 7). These manifestations not only impact on their academic performance and overall wellbeing, but also increase suicide risk. In light of this, early identification is of particular importance. Presently, the assessment and monitoring of depression in this population predominantly depend on subjective psychological scales. Most of these scales depend on individuals' self-reported emotional states, which easily cause ignored and missed (5, 8, 9). Therefore, there is an imperative to develop facile and effective methodologies that can offer objective and accurate identification of depression, facilitating large-scale screening on Chinese university campuses.

The acoustic approach offers distinct advantages for depression assessment by addressing limitations of conventional methods. As objective physiological measures, acoustic features circumvent the response biases inherent in self-report instruments while capturing subtle emotional cues through prosodic variations such as pitch variability and speech rhythm. Acoustic analysis provides multidimensional insights by concurrently revealing emotional states through prosodic characteristics and physiological changes via glottal features including vocal fold vibration patterns. These features encompass a range of quantitative data extracted from speech signals, such as pitch, speech rate, volume, timbre, and elements related to speech pauses and fluency. Meanwhile, acoustic features can provide insights into the speaker's physical health, emotional fluctuations, and psychological traits (10). According to the linear speech production system, acoustic features include spectral, prosodic, and glottal features (11, 12). Spectral features represent the correlation between changes in vocal tract shape and movements of articulatory organs, reflecting the characteristics of speech signals in the frequency domain (13). Prosodic features can be characterized by rhythm, intensity, pitch, and duration, which correspond to the elements of stress, timing, and intonation in speech (14–16). Glottal features provide insights into the type of phonation and vocal quality conveyed by irregular sounds, reflecting the airflow from the lungs through the glottis and the vibrations of the vocal folds (13). These acoustic features have been employed in the domain of speech emotion recognition. Prior studies have identified that individuals with depression exhibit distinct acoustic features, characterized by decreased vocal volume, a reduced pitch range and voice intensity, a slower speech rate, prolonged pauses, and a monotonous tone (17–21). Variations in emotional states and fatigue can influence muscle tension, leading to pronunciation errors and alterations in vocal tract characteristics (22). Cognitive impairments may hinder the planning and execution of neuromuscular commands essential for voice production (23, 24). Most importantly, voice-based methods enable real-time, scalable screening without active user participation. Therefore, acoustic features, as objective, readily accessible, non-invasive physiological measures, have been increasingly utilized in the study of depression (10, 25–28).

Notably, the direct extraction and analysis of acoustic features using conventional statistical methods can be quite complex, potentially resulting in diminished recognition performance (29). The advantages of machine learning methods lie primarily in their exceptional modeling flexibility and algorithmic scalability. Compared to traditional statistical methods, machine learning can effectively capture complex nonlinear relationships and interaction effects between variables through automated feature learning mechanisms, particularly excelling in handling high-dimensional feature spaces and unstructured data (such as medical images, natural language text, etc.). In terms of model optimization, machine learning significantly enhances generalization performance through ensemble learning frameworks, regularization constraints, and rigorous cross-validation strategies. This approach overcomes the strict reliance of traditional statistical methods on linear assumptions and specific distribution patterns, thereby demonstrating stronger adaptability in modeling complex real-world problems. By automatically learning from data, machine learning algorithms are capable of establishing functional relationships, identifying latent patterns, and generating predictions that were previously inaccessible via conventional statistical methodologies (30). This capacity is particularly crucial for the early detection and intervention of depression, as the timely identification of symptoms can profoundly influence management and intervention strategies for university students. Several machine-learning techniques such as Support Vector Classification (SVC), Random Forest (RF), Light Gradient Boosting Machine (LightGBM), Linear Discriminant Analysis (LDA), and logistic regression (LR) have been applied for classification. Among these methods, SVM is particularly adept at finding hyperplanes that best separate different classes in high-dimensional spaces, making it a powerful tool for classification tasks (31). RF constructs an ensemble of decision trees and merges their outputs to enhance predictive accuracy (32, 33). LightGBM utilizes a histogram-based approach to bin continuous features, thereby substantially accelerating the training process while preserving high accuracy (10, 34). LR is distinguished by its simplicity and physical interpretability (35). LDA can handle high-dimensional data, and effectively separate classes by maximizing the ratio of between-class variance to within-class variance. This characteristic allows LDA as an effective tool for classification tasks. Although machine learning has the potential to aid in the detection of depression, these models often function as a “black box” that requires further interpretation (36). Correspondingly, SHapley Additive exPlanations (SHAP) is a well-established *post-hoc* interpretability method that ranks selected features according to their contributions, with larger values indicating a greater contribution (37). Most acoustic studies on depression primarily focus on group differences and employ one or more machine learning techniques (13, 38–40), the integration of multiple machine learning models with SHAP interpretability analysis among Chinese university students is rarely reported.

This study aims to present an intelligent system that not only identifies depression through acoustic features but also integrates advanced machine learning techniques to improve detection accuracy and reliability. We sought to address the following questions: First, do acoustic features exhibit alterations in depression among Chinese university students? Second, how do these acoustic alterations correlate with depression severity in the speech of university students? Third, can acoustic features effectively differentiate between depression and non-depression using machine learning methodologies? If this is the case, which acoustic features hold relative importance in the classification of depression? The integration of these methodologies could facilitate the development of more effective strategies for monitoring and intervening in depression on Chinese university campuses.

## 2 Materials and methods

### 2.1 Participants

**Figure 1:**
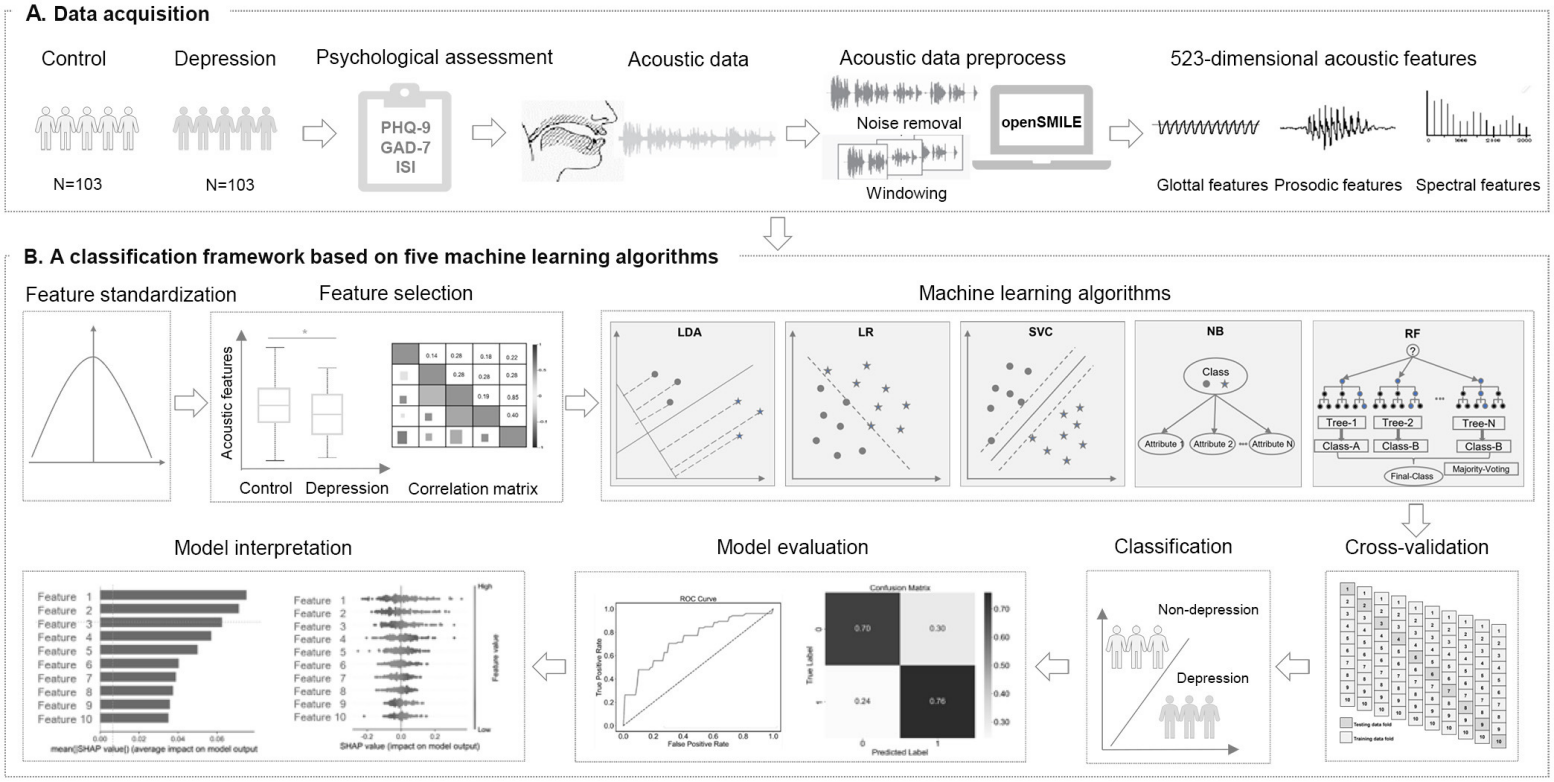
Study design. **(A)** A total of 206 Chinese university students were recruited for this cross-sectional study. We collected psychological questionnaires and acoustic data through a WeChat official account platform. After data preprocessing, we extracted 523–dimensional acoustic features for all participants. **(B)** *Z* score normalization was applied to the data. Acoustic features with significant differences were selected, and correlation analysis was performed. Subsequently, five machine learning-based classification models were built using 10-fold cross-validation. Finally, we evaluated the performance of models and assessed the importance of the selected acoustic features.

This cross-sectional study was carried out at Xinxiang Medical University between March and May 2024. A total of 206 university students (103 subjects with depression and 103 matched controls) were recruited for this study (Figure 1). University students with depression were included if: (1) between 17 to 26 years old; (2) Patient Health Questionnaire-9 (PHQ-9) scores≥5; (3) able to read and understand Chinese; (4) have not received minimally adequate treatment (antidepressant medication, neurostimulation therapy, and evidence-based psychotherapy). Controls were required to meet all of the following criteria: (1) PHQ-9 scores < 5; (2) Generalized Anxiety Disorder-7 (GAD-7) scores < 5; (3) Insomnia Severity Index (ISI) scores < 8; and (4) no personal or family history of mental disorders. All subjects were excluded if: (1) history of mental disorder or drug abuse; (2) history of neurological disorders; (3) primary language other than Chinese. All participants provided written informed consent approved by the institutional review boards of the Second Affiliated Hospital of Xinxiang Medical University (XEEFY-2023-35-4), in accordance with the Declaration of Helsinki's Ethical Principles of Medical Research Involving Human Subjects.

### 2.2 Psychological assessment

All psychological questionnaires were completed on the WeChat-based official account platform. Depression was assessed by the self-rated PHQ-9, which is a widely used depression detection instrument based on Diagnostic and Statistical Manual of Mental Disorders, fourth edition (DSM-IV) criteria in primary care evaluation (41). In this study, a PHQ-9 total score ≥ 5 was defined as depression (42). Severity of anxiety was assessed by the GAD-7, and sleep by the ISI. To address potential biases in PHQ-9 self-assessment, we implemented a comprehensive quality control protocol. All participants submitted questionnaires through an encrypted WeChat platform, ensuring anonymity and reducing social desirability bias. Field investigators received standardized training to supervise data collection consistently. Post-collection, two researchers independently reviewed the data, removing duplicate submissions, logical inconsistencies (e.g., extreme age values or uniform responses), and ambiguous answers (e.g., “don't know”). For minimal missing data, multiple imputation was applied to maintain data completeness. These measures ensured the reliability and validity of our findings.

### 2.3 Acoustic data acquisition

The acoustic data were recorded in a quiet room with minimal background noise on the day of psychological assessment. Acoustic data was collected through a WeChat official account platform. All participants read the neutral text “Let life be beautiful like summer flowers” displayed on the screen for about 3 min. To ensure the continuity of psychological state and voice data, all participants transitioned immediately to a standardized recording environment for audio collection after completing the mental health questionnaire. The recordings were conducted in the same room under the guidance of trained investigators, ensuring a relatively quiet environment to minimize reverberation and other environmental noise influences. We standardized the recording equipment by using HUAWEI MatePad BAH3-AN10 tablets. This consistency ensures standardized recording quality and reduces the impact of environmental noise. Each participant was asked to read same text at a normal speech rate and intonation, maintaining approximately 20 cm distance from the device's microphone to ensure data quality. To reduce possible confounding factors, a set of pre-processing steps were taken, our audio preprocessing pipeline is split into two stages: voice activity detection (VAD) and resampling:

(1) voice activity detection (VAD): Non-vocal parts has been removed to reduce interference with subsequent investigations. We used a dual-threshold endpoint detection algorithm to cut and retain relevant sections. This algorithm operates by evaluating the short-term energy and zero-crossing rate of the audio signal. The implementation sequence of the algorithm is outlined as follows: Initially, we denote s(*n*) as the input signal, where *n* represents the time index. Then, we calculate the short-time energy (STE) using a Hamming window, denoted as w(*n*). The STE can be determined as:


(1) $$E(n)\;=\;\sum_{m=-\infty }^{+\infty }s^{2}\left(m\right)\cdot w\left(n-m\right)$$

The start and end of speech segments can be approximated as:


(2) $$\begin{array}{l} n_{\text{start}}\;=\begin{array}{c} \min\\ n \end{array}\left(n|\left(n\right)>H\text{T }\right),\\ n_{\text{end}}\;=\begin{array}{c} \min\\ n>n_{start} \end{array}\left(n|\left(n\right)<LT\right) \end{array}$$

Where *HT* and *LT* are the high and low thresholds, respectively. The high threshold *HT* is determined based on the peak energy levels typical of vocal segments to ensure accurate detection of speech onset, while the low threshold *LT* is set to identify the trailing edges of speech, avoiding the inclusion of minimal non-speech artifacts. This can minimize the possibility of false positives and negatives. The level of accuracy is particularly crucial in depression detection, as the vocal features such as tone, pitch, and speech pauses play a significant role in diagnosis. Additionally, the merits of employing VAD in our study include the improved clarity of the extracted vocal features. By isolating pure speech segments, our approach ensures that the subsequent feature analysis is not contaminated by background noise or silence, thus enhancing the predictive power of our machine learning approach.

(2) Resampling: Participants' recordings were initially stored with a sampling rate of 48 kHz and a bit rate of 128 kbps. Upon uploading, the audio file was compressed to 8 kHz and 5.6 kbps. For subsequent analysis, we resampled the audio to a unified 44.1 kHz sampling rate. This rate is frequently used in speech signal analysis due to its compliance with the Nyquist Theorem, effectively capturing the pertinent frequency range of human speech while enhancing computational efficiency. This strategic choice ensures our model's applicability across various recording environments, thus broadening the potential for real-world deployment.

#### 2.3.1 Signal-to-noise ratio (SNR) calculation

To ensure audio clarity and validate recording quality, we calculated the SNR for each voice sample as follows:

(1) Signal preprocessing: Audio recordings were resampled to 16 kHz (mono channel) and normalized to 16-bit PCM format. (2) Noise reference extraction: Non-speech segments (e.g., silence, background noise) were detected using WebRTC Voice Activity Detection (VAD) in aggressive mode (level = 3), with 30-ms frame segmentation. (3) Power calculation: Speech power (*P*_signal)_ was computed from VAD-identified speech segments. Noise power (*P*_noise_) was derived from non-speech segments. SNR formula: SNR_dB_ = 10·log_10_($\frac{P_{signal}-P_{noise}}{P_{noise}}$). (5) Quality control: Recordings with SNR < 20 were excluded from analysis. The final dataset exhibited a mean SNR of 25.06 dB (SD = 3.33).

### 2.4 Acoustic feature extraction

Subsequently, we extracted acoustic features from the voice samples using the emoLarge feature set provided by the open-source toolkit called Speech and Music Interpretation by Large-space Extraction (openSMILE v.3.0.1) and the Librosa toolkit (43). Acoustic features were categorized into Low-Level Descriptors (LLD) and High-Level Statistical Functions (HSF). LLD represents the basic attributes of the speech signals. In this study, acoustic features were grouped into three main categories: spectral, prosodic and glottal features. Specifically, spectral features refer to Mel-frequency cepstral coefficients (MFCC). Prosodic features include Fundamental Frequency (F0), Effective segmentation (Duration), Sound Pressure Level (SPL), voiceless, voiced, Short-Time Energy (STE), Zero-Crossing Rate (ZCR), and Energy. Glottal feature consists of Formant Frequencies F1, F2, F3, Formant Bandwidths B1, B2, B3, jitter, and shimmer. HSF is descriptive statistical analysis of the LLDs, including maximum, minimum, mean, range, standard deviation, kurtosis, and skewness. Among these features, MFCC features were particularly emphasized due to their computational simplicity and significant discriminative power (43). The extraction of MFCC features generally involves several steps: pre-emphasis, framing, windowing, fast fourier transform, Mel filter bank, logarithmic computation, discrete cosine transform, and the extraction of dynamic differential parameters (44). Ultimately, 523-dimensional acoustic features were extracted for each participant. All acoustic features were divided into three main categories: 273 spectral features, 120 prosodic features, and 130 glottal features.

### 2.5 Statistical analysis

The Kolmogorov-Smirnov single-sample test was applied to assess the normality of continuous variables, confirming the normal distribution of all subjects. Continuous data were demonstrated as mean ± standard deviation or median, and ranges, and compared using two-tailed Student's *t* tests, or Mann-Whitney *U-*test, respectively. Categorical data were reported as frequencies (%), and comparisons were performed with the chi-square test. Partial correlation analyses were conducted, controlling for age and gender as covariates. *P* < 0.05 was considered statistically significant. Statistical analysis was performed using SPSS 23.0 software.

### 2.6 Machine learning algorithms for classification

In this study, we employed five supervised machine learning techniques to develop classifiers, specifically Support Vector Classification (SVC), Random Forest (RF), Linear Discriminant Analysis (LDA), and Naive Bayes (NB). Prior to model training, *Z*-score normalization was implemented to mitigate the influence of data units, expedite model convergence, and minimize biases among features, thereby enhancing model accuracy and efficiency. For feature selection, we posited that acoustic features exhibiting significant variations and associations with depression may possess superior discriminative capabilities. In this study, we employed five supervised machine-learning techniques to construct the classifiers: SVC, RF, LDA, and NB. Prior to model training, *Z*-score normalization was implemented to mitigate the influence of data units, accelerate model convergence, and minimize biases among features, thereby improving the accuracy and efficiency. For feature selection, we hypothesized that acoustic features exhibiting significant changes and associations with depression may have superior discriminative ability. In this study, we employed two-tailed Student's *t-*t*es*ts and Pearson correlation analyses to identify statistically significant acoustic features for input. A ten-fold cross-validation approach, allocating 90% of the data for training and 10% for internal validation, was implemented to optimize the model and mitigate overfitting and bias. The GridSearchCV method was utilized to determine the optimal hyperparameters for five machine learning models, while other hyperparameters were set as default.

Specifically, the optimized parameters were solvers (“lbfgs”, “liblinear”, “saga”) and shrinkage (None, “auto”, “log”, 0.1, 0.5, 1.0) in LDA, regularization parameter C (0.1, 1, 10, 100), penalty (None, “l2”) and solvers (“lbfgs”, “liblinear”, “saga”) in LR, regularization parameter C (0.1, 1, 10, 100) and kernels (“linear”, “rbf”, “poly”) in SVC, variance smoothing (1e-9, 1e-8, 1e-7, 1e-6) in NB, and the number of trees in the forest (n_estimators: 10, 100, 200, 500, 1000) and the maximum depth of the trees (max_depth: None, 5) in RF. Subsequently, five classifiers were utilized to construct the depression classification model. Depression was classified as binary, with a PHQ-9 score of ≥ 5 indicating the presence of depression (absent depression = 0, and depression = 1). Evaluation metrics of the models included area under the receiver operating characteristic curve (AUC), ROC (Receiver Operating Characteristic), accuracy, precision, recall, and F1 score. SHAP analysis was utilized to enhance the interpretability of five machine learning models. The contribution and impact of the selected features were assessed using SHAP values. The ten most significant acoustic features were identified and visualized utilizing the SHAP Python package. All machine learning procedures were implemented using the python sklearn package version 1.2.1 (https://scikit-learn.org/).

## 3 Results

### 3.1 Demographic characteristics

The normal distribution of data was tested using the Shapiro–Wilk *W*-test (*P* > 0.05). Compared to controls, depression group showed higher scores on the PHQ-9, GAD-7 score, and ISI score (*P* < 0.05). No significant differences were identified in age, gender, or education level between groups (*P* > 0.05). See Table 1.

**Table 1:** Demographic characteristics of controls and depression among Chinese university students.

**Characteristics**	**Control (*N* = 103)**	**Depression (*N* = 103)**	**Statistics**	***P* values**
Age, year	20.99 ± 1.82	20.58 ± 1.58	1.718	0.087
Gender (Female/Male)	75/28	75/28	0.000	1.000
Education level (Year)	14.93 ± 1.65	14.57 ± 1.37	1.603	0.111
PHQ-9 score	1.59 ± 1.35	8.18 ± 3.38	−18.388	< 0.001^*^
GAD-7 score	0.85 ± 1.22	5.50 ± 3.78	−11.853	< 0.001^*^
ISI score	2.24 ± 1.95	7.15 ± 4.30	−11.080	< 0.001^*^

Continuous data are presented as mean (SD) and categorical data as n (%). ^*^Significance level was set at P < 0.05. GAD-7, Generalized Anxiety Disorder-7; ISI, Insomnia Severity Index; PHQ-9, Patient Health Questionnaire-9.

### 3.2 Significant changes of acoustic features in depression

**Figure 2:**
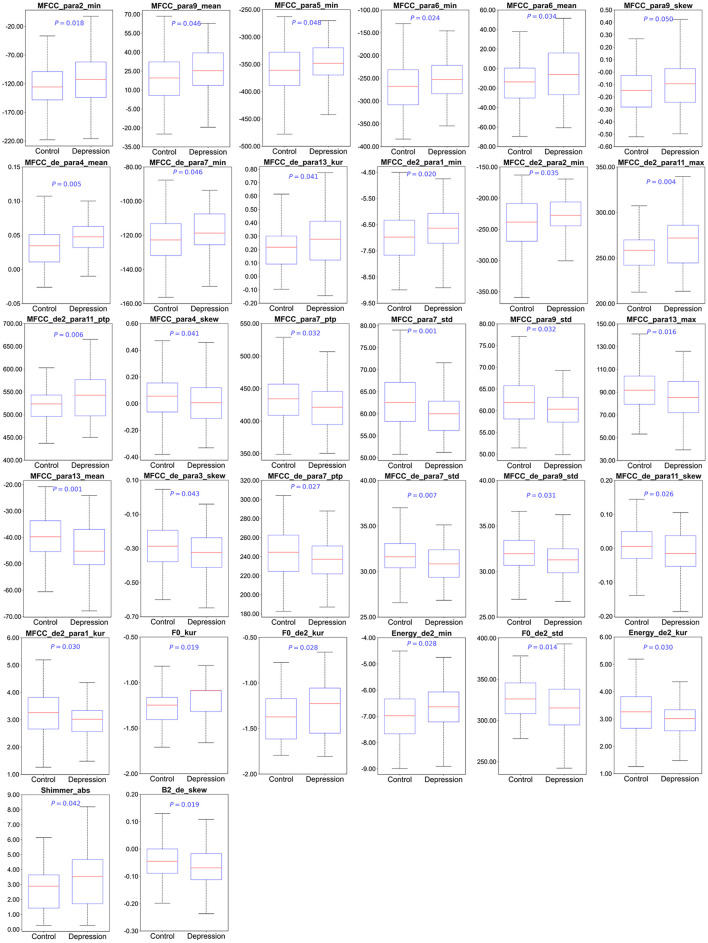
The differences in the acoustic features between control and depression among Chinese university students. F0, Fundamental Frequency; MFCC, Mel Frequency Cepstral Coefficients. Significance level was set at *P* < 0.05.

Compared to the control group, 32 differentially acoustic features were identified in the depression group. Specifically, 25 spectral features, 5 prosodic features, and 2 glottal features were significantly altered according to three categories. The detailed results are summarized in Table 2 and Figure 2.

**Table 2:** Significant differences of acoustic features between control and depression. among Chinese university students.

**Categories**	**Acoustic features**	**Control**	**Depression**	**Statistics**	***P* values**
**Spectral features**					
	MFCC_para2_min	−125.665 ± 36.722	−112.619 ± 41.713	−2.382	0.018
	MFCC _para9_mean	19.562 ± 21.069	25.332 ± 20.114	−2.010	0.046
	MFCC _para5_min	−360.949 ± 47.296	−348.195 ± 44.763	−1.988	0.048
	MFCC _para6_min	−267.82 ± 50.571	−252.729 ± 44.179	−2.281	0.024
	MFCC _para6_mean	−13.698 ± 25.677	−6.073 ± 25.565	−2.136	0.034
	MFCC _para9_skew	−0.147 ± 0.183	−0.094 ± 0.205	−1.973	0.050
	MFCC_de_para4_mean	0.035 ± 0.036	0.048 ± 0.031	−2.832	0.005
	MFCC_de_para7_min	−122.761 ± 14.222	−118.73 ± 14.527	−2.012	0.046
	MFCC_de_para13_kur	0.218 ± 0.185	0.278 ± 0.233	−2.053	0.041
	MFCC_de2_para1_min	−6.976 ± 1.197	−6.635 ± 0.859	−2.345	0.020
	MFCC_de2_para2_min	−238.468 ± 41.094	−227.644 ± 31.426	−2.123	0.035
	MFCC_de2_para11_max	258.389 ± 28.661	271.946 ± 37.433	−2.918	0.004
	MFCC_de2_para11_ptp	523.45 ± 43.036	542.494 ± 55.498	−2.752	0.006
	MFCC _para4_skew	0.056 ± 0.167	0.007 ± 0.174	2.061	0.041
	MFCC _para7_ptp	434.236 ± 46.834	421.327 ± 38.34	2.165	0.032
	MFCC _para7_std	62.567 ± 5.636	59.983 ± 4.863	3.523	0.001
	MFCC _para9_std	61.899 ± 5.802	60.36 ± 4.324	2.159	0.032
	MFCC _para13_max	91.773 ± 17.773	85.359 ± 20.023	2.431	0.016
	MFCC _para13_mean	−39.789 ± 9.172	–45.248 ± 13.401	3.411	0.001
	MFCC_de_para3_skew	−0.287 ± 0.134	−0.324 ± 0.13	2.036	0.043
	MFCC_de_para7_ptp	244.541 ± 24.687	237.238 ± 22.247	2.231	0.027
	MFCC_de_para7_std	31.623 ± 2.167	30.854 ± 1.862	2.733	0.007
	MFCC_de_para9_std	31.959 ± 2.468	31.283 ± 1.978	2.170	0.031
	MFCC_de_para11_skew	0.006 ± 0.068	−0.015 ± 0.063	2.247	0.026
	MFCC_de2_para1_kur	3.262 ± 0.896	3.018 ± 0.689	2.190	0.030
**Prosodic features**					
	F0_kur	−1.248 ± 0.26	−1.05 ± 0.809	−2.370	0.019
	F0_de2_kur	−1.373 ± 0.29	−1.227 ± 0.6	−2.221	0.028
	Energy_de2_min	−6.976 ± 1.197	−6.635 ± 0.859	−2.345	0.020
	F0_de2_std	326.206 ± 25.276	315.237 ± 37.086	2.480	0.014
	Energy_de2_kur	3.262 ± 0.896	3.018 ± 0.689	2.190	0.030
**Glottal features**					
	Shimmer_abs	2.888 ± 2.026	3.534 ± 2.485	−2.045	0.042
	B2_de_skew	−0.045 ± 0.076	−0.069 ± 0.069	2.371	0.019

F0, Fundamental Frequency; MFCC, Mel Frequency Cepstral Coefficients.

### 3.3 Correlation between acoustic features and depression severity

**Figure 3:**
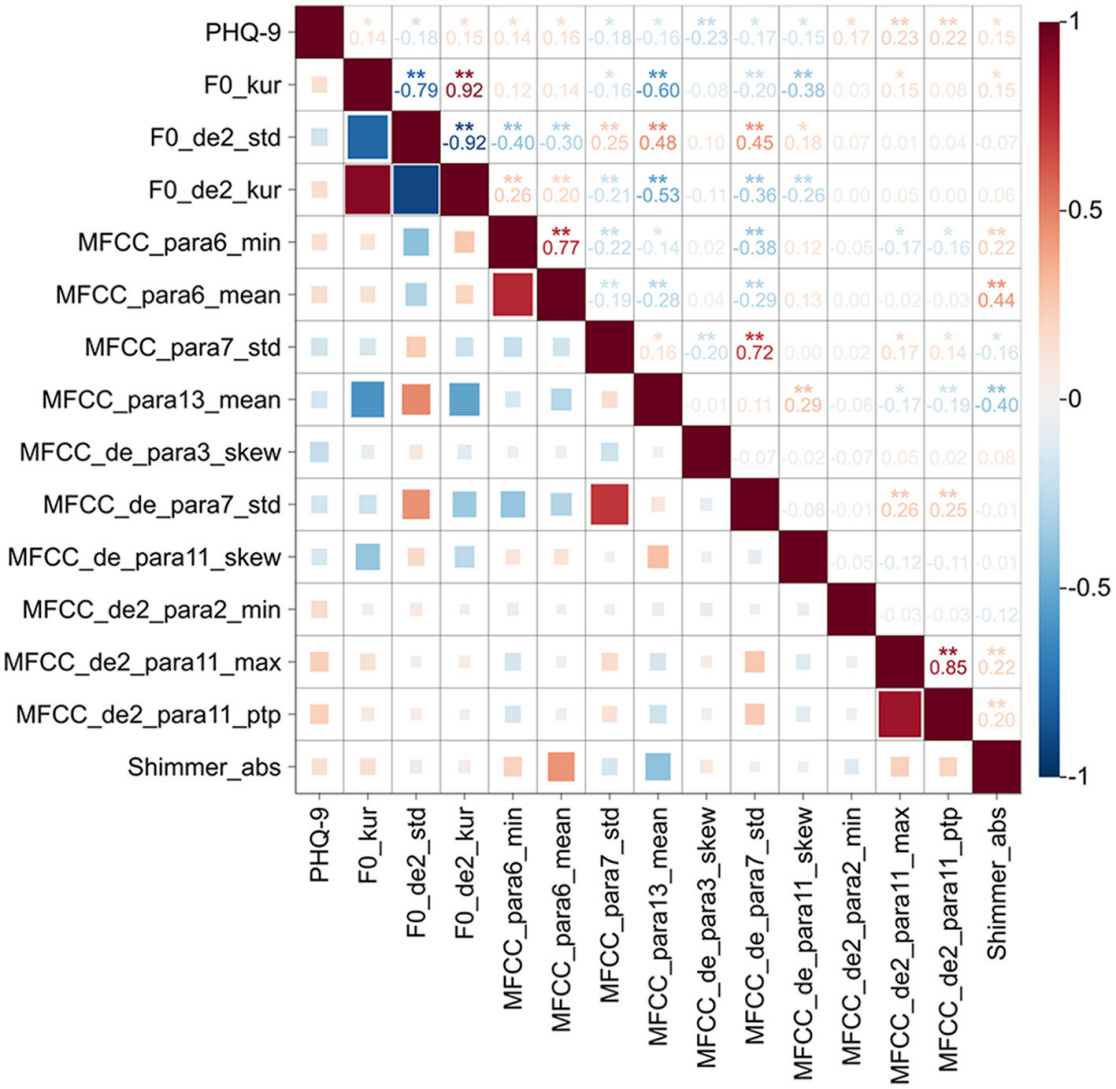
Correlation of acoustic features and PHQ-9 scores in depression group. Heatmap of the correlations between acoustic features and PHQ-9 scores. The horizontal and vertical axes represent the PHQ-9 score and acoustic features. Red and blue indicate positive and negative correlations, respectively. In the upper right of the heatmap, the correlation coefficients between the two metrics are labeled, and statistical significance is shown by ^*^P < 0.05; ^**^P < 0.01. Color bar represents the intensity of the correlations.

A total of 10 spectral features, 3 prosodic features, and 1 glottal features significantly associated with PHQ-9 scores in depression group. Specifically, PHQ-9 scores was positively correlated with spectral features (MFCC_para6_min, MFCC _para6_mean, MFCC _de2_para2_min, MFCC _de2_para11_max, MFCC _de2_para11_ptp), prosodic features (F0_kur, F0_de2_kur, and F0_de2_std) and glottal feature (Shimmer_abs), while negatively correlated with spectral features (MFCC _para7_std, MFCC _para13_mean, MFCC _de_para3_skew, MFCC _de_para7_std, MFCC _de_para11_skew), and prosodic feature (F0_de2_std). The detailed results are presented in Table 3 and Figure 3.

**Table 3:** Correlation between acoustic features and PHQ–9 scores in depression group.

**Categories**	**Acoustic features**	***R* values**	***P* values**
**Spectral features**			
	MFCC_para6_min	0.148	0.035^*^
	MFCC _para6_mean	0.150	0.032^*^
	MFCC _de2_para2_min	0.136	0.052
	MFCC _de2_para11_max	0.230	0.001^**^
	MFCC _de2_para11_ptp	0.223	0.001^**^
	MFCC _para7_std	−0.195	0.005^**^
	MFCC _para13_mean	−0.163	0.020^*^
	MFCC _de_para3_skew	−0.222	0.001^**^
	MFCC _de_para7_std	−0.191	0.006^**^
	MFCC _de_para11_skew	−0.159	0.023^*^
**Prosodic features**			
	F0_kur	0.140	0.046^*^
	F0_de2_kur	0.156	0.026^*^
	F0_de2_std	−0.200	0.004^**^
**Glottal features**			
	Shimmer_abs	0.142	0.042^*^

F0, Fundamental Frequency; MFCC, Mel Frequency Cepstral Coefficients; PHQ–9, Patient Health Questionnaire–9. ^*^P < 0.05, ^**^P < 0.01.

### 3.4 Classification results

**Figure 4:**
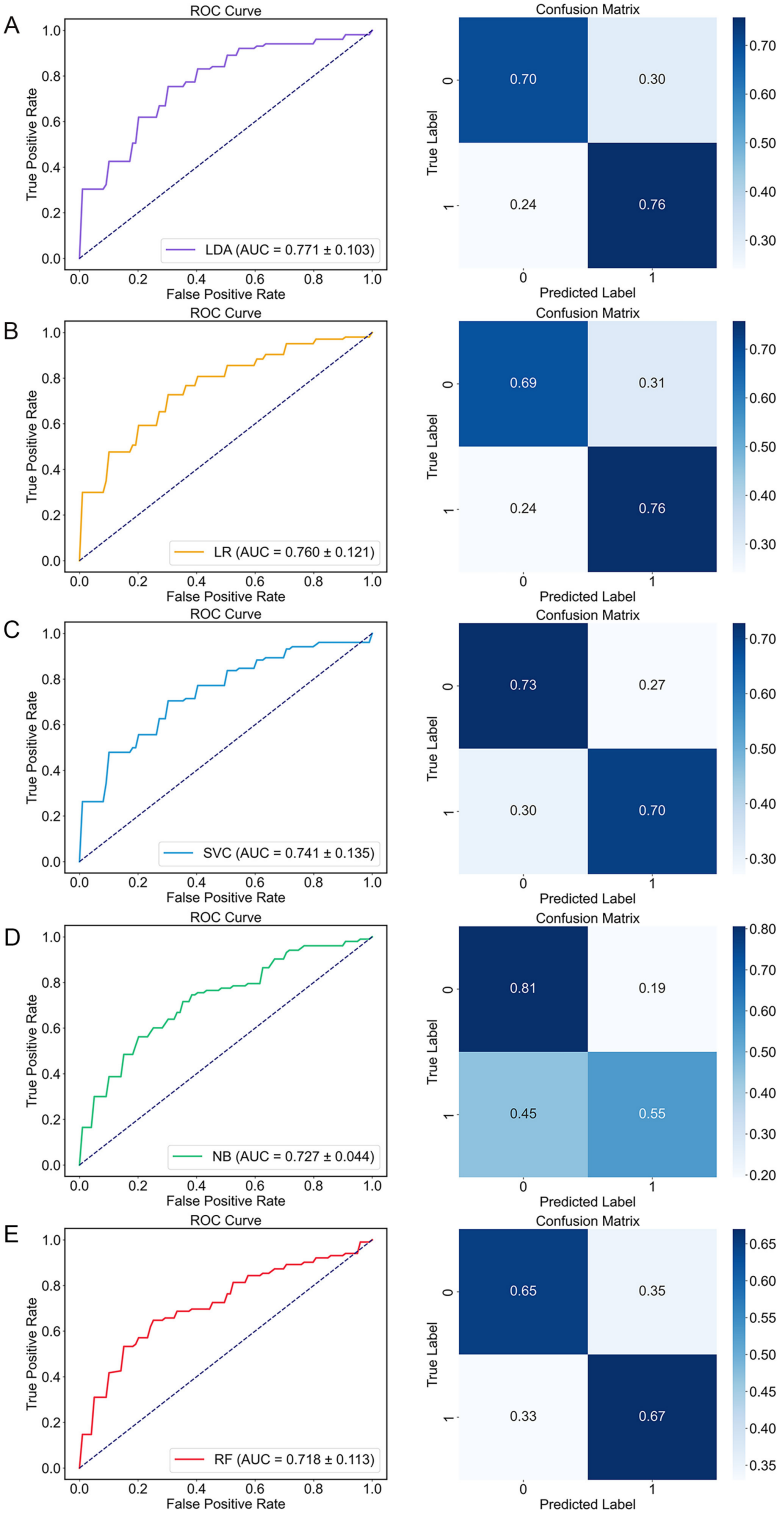
The performance of five machine learning algorithms. Receiver operating characteristic (ROC) curve (left) and confusion matrix (right). **(A)** LDA; **(B)** LR; **(C)** SVC; **(D)** NB; **(E)** RF. AUC, area under the curve; LDA, Linear Discriminant Analysis; LR, Logistic Regression; NB, Naive Bayes, RF, Random Forest; ROC, Receiver operating characteristic; SVC, Support Vector Classification.

**Figure 5:**
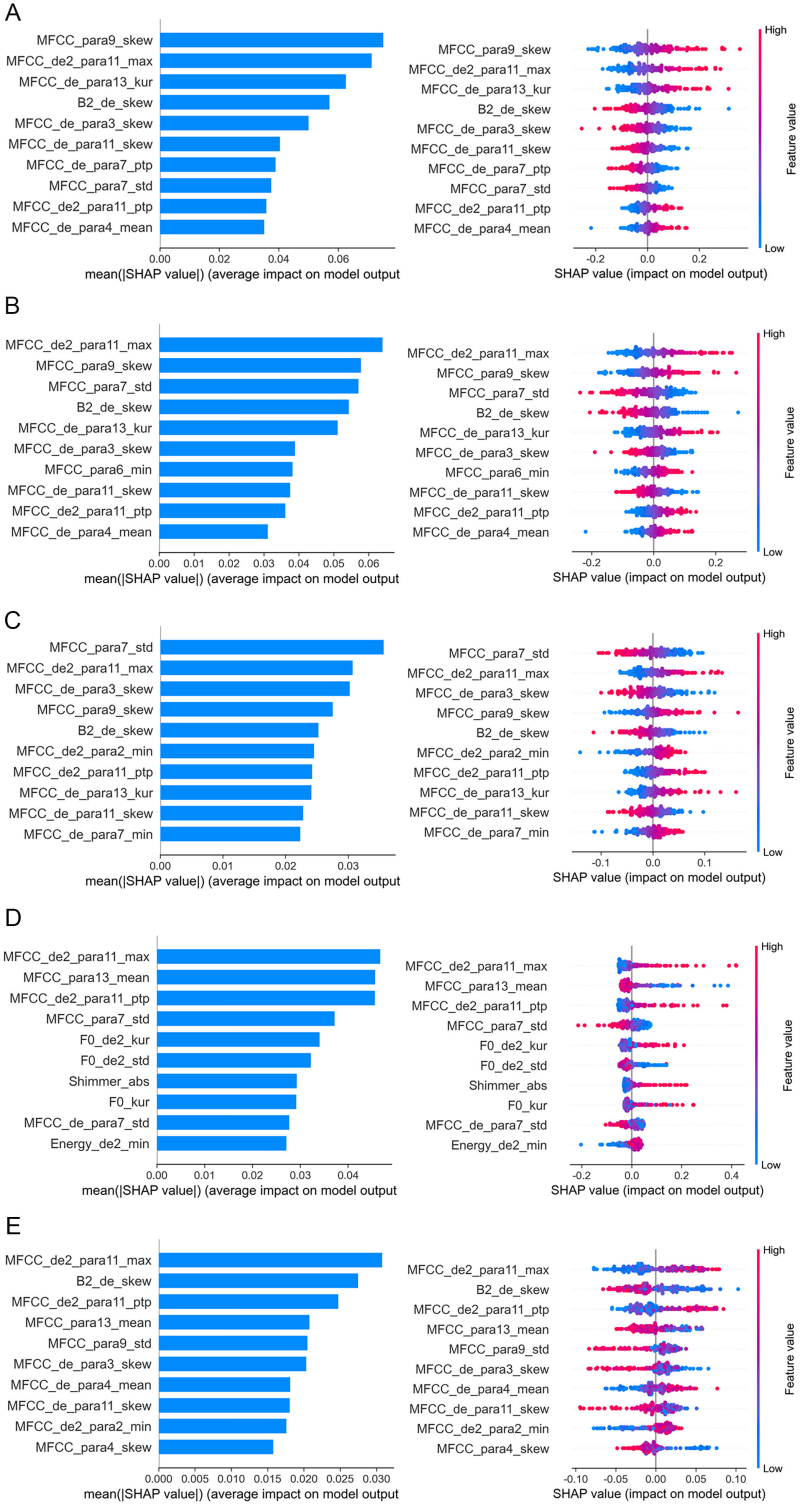
Feature importance analysis based on SHAP method. The top 10 acoustic features identified by SHAP for the classification model are ordered from most to least important. **(A)** LDA; **(B)** LR; **(C)** SVC; **(D)** NB; **(E)** RF. In the left plots, feature importance is determined by calculating the mean of absolute SHAP values for each feature. A bar plot displays the mean absolute SHAP value for the top 10 features, where larger bars indicate the feature's importance in discriminating between depression and non-depression. In the right plots, each dot corresponds to the SHAP value of each sample. Red and blue correspond to higher and lower values, respectively. LDA, Linear Discriminant Analysis; LR, Logistic Regression; NB, Naive Bayes; RF, Random Forest; SHAP, Shapley Additive Explanations; SVC, Support Vector Classification.

Based on the above results, acoustic features with significant alterations were selected as input for the machine learning algorithm. As shown in Table 4 and Figure 4, LDA model achieved superior classification performance compared with SVC, RF, NB, and LightGBM. Its trained 10-fold cross-validated classifier had an accuracy of 72.8% with an AUC of 0.771 in distinguishing depression from controls. LR model had the second highest accuracy of 72.3% with an AUC of 0.76. In contrast, RF exhibited the lowest performance (AUC = 0.718, Accuracy = 0.66, Precision = 0.665, Recall = 0.67, and F1 = 0.664). The top ten acoustic features that had the most influence on prediction of depression were identified using the SHAP method. The SHAP importance plots of five models is shown in Figure 5, which shows how high and low values of each feature are related to SHAP. Consistently, We found that MFCC was the most important feature among five machine learning models. MFCC_de2_para11_max, and MFCC_para7_std, and MFCC_para9_skew had the highest mean absolute SHAP value in the LDA, LR, SVC, NB, and RF models, respectively.

**Table 4:** Performances of five machine learning algorithms using acoustic features.

**Classifier**	**AUC**	**Accuracy**	**Precision**	**Recall**	**F1 score**
LDA	0.771	0.728	0.735	0.757	0.737
LR	0.760	0.723	0.730	0.757	0.734
SVC	0.741	0.715	0.732	0.700	0.710
NB	0.727	0.680	0.737	0.553	0.627
RF	0.718	0.660	0.665	0.670	0.664

AUC, area under the curve; LDA, Linear Discriminant Analysis; LR, Logistic Regression; NB, Naive Bayes, RF, Random Forest; SVC, Support Vector Classification.

## 4 Discussion

In this study, we developed an acoustic-based system for identifying depression among Chinese university students by integrating 3-min voice recordings with five machine learning algorithms. Firstly, individuals with depression demonstrated significant alterations in spectral, prosodic, and glottal features compared to controls. Secondly, these three categories of acoustic features were significantly correlated with the severity of depression. Lastly, these distinct acoustic variations were employed to construct classification models for detecting depression in university students. LDA model exhibited optimal performance, with a mean AUC of 0.771 and an accuracy rate of 0.728. An analysis of feature importance indicated that MFCC features were the most significant contributors to the model's classification efficacy. This research highlights the potential of acoustic features as an objective measure for identifying and characterizing depression among Chinese university students. The findings suggest that MFCC features combined with LDA classifier could provide a more objective and accurate tool to complement current assessments.

A significant finding of the study is the presence of acoustic differences between individuals with depression and control group, specifically in spectral, prosodic, and glottal features. Spectral features represent the characteristics of speech signals within the frequency domain and demonstrate the relationship between alterations in vocal tract shape and the occurrence of motion (13). These features are highly highly dependent on speech content. A prior study constructed a set of 3 (emotion) × 4 (task) speech scenarios involving 104 participants, revealing that spectral features significantly differed between individuals with and without depression (45). Variations in prosodic features primarily reflect speaking behavior in response to stress, intonation, and emotional factors. Notably, F0 and energy indicate the pitch and loudness of speech (15). The depression group demonstrated significantly lower F0_de2_std and Energy_de2_kur values, suggesting that individuals with depression exhibit reduced pitch variability, which may manifest as a more monotonous tone. Mundt et al. (38) identified a reduction in F0 during depressive states, indicative of alterations in the vocal cord vibration cycle. According to the source-filter theory of speech production, glottal features represent the initial sound source, which is subsequently modified by the vocal tract. The characteristics of the glottal pulse and its shape are crucial in the acoustic analysis of depression. Depression group demonstrated significantly elevated Shimmer_abs values, suggesting heightened irregularities in voice intensity. These differences illustrate the potential physiological impact of depression on voice production mechanisms. This impact may manifest as delayed planning and preparation processes for neuromuscular commands, attributable to cognitive impairments, as well as alterations in emotional states that influence muscle tension, resulting in articulation errors and alterations in vocal tract characteristics. Collectively, our study provides further evidence of cross-sectional acoustic variations in university students experiencing depression. The use of non-invasive and more accessible vocal information for preliminary screening purposes holds promise for reducing the costs of psychological assessments in school.

Another notable finding is significant association between spectral, prosodic, and glottal features and depression in Chinese university students. These three feature categories are linked to perceptual and physiological components that characterize by human speech production model. Spectral features were associated with fewer vocal tract alterations in depression due to the tighter vocal tract caused by slow thinking, reduced language communication and activity (40). Research has shown that prosodic features can serve as a significant indicator of depression severity. A recent study involving 57 participants demonstrated that naive listeners were capable of perceiving the severity of depression through vocal recordings, with vocal prosody explaining a significant portion of the variance in depression scores (46). Prior research has similarly identified a significant correlation between F0 and the severity of depression (38, 47). Depression induces atypical alterations in the autonomic nervous and somatic systems, potentially influencing muscle tension and respiratory rate, which consequently result in alterations to glottal features. Our findings align with prior research, further confirming the association between acoustic features and depression. Therefore, we speculate that depression may exert a direct effect on the physiological structures underlying vocal production—the vocal tract, larynx, and lungs, thereby altering the voice structure itself, and ultimately manifesting as spectral, prosodic, and glottal feature changes.

More interestingly, our study demonstrated that university students experiencing depression could be effectively differentiated from control subjects through the application of rapid, cost-efficient, feasible, and automated speech-based methodologies. Among five machine learning models, LDA exhibited the highest performance, achieving an AUC of 0.771 and accuracy of 0.728. The primary advantage of LDA lies in its capacity to provide an effective and interpretable classification method by maximizing the ratio of between-class variance to within-class variance. This allows LDA to generate a linear combination of features that optimally differentiates between distinct classes, making it particularly useful in contexts where classes are well-separated within the feature space. In line with our findings, Kaur et al. (48) proposed a two-phase speech-based depression detection system and reported that LDA outperformed K-Nearest Neighbors (KNN), SVC, and LR classifiers, achieving a superior F1-score of 0.846. Andreev et al. (49) applied LDA on data from 35 individuals with depression and 50 controls to distinguish between the two groups, utilizing functional networks' global networks. They also found LDA achieved the optimal performance, with a classification accuracy exceeding 0.6. Ji et al. (50) reported that LDA can be utilized for voice analysis of depression detection, attaining an accuracy of 78.9%. These findings highlight the robustness of the LDA model in handling classification tasks, suggesting its potential practical applications in future research. Collectively, the integration of five distinct machine learning methodologies in this study facilitates the development of more effective and robust classification frameworks. These frameworks can be specifically tailored to acoustic datasets, thereby enhancing predictive performance in educational applications.

Perhaps the most compelling finding is that MFCC features contributed the most to five model's classification efficacy. To elucidate the decision-making processes of these models, we employed SHAP for model interpretation. This makes our classification model more interpretable and ultimately makes the model suitable for applications. Consistently, our analysis identified MFCC features (MFCC_de2_para11_max, and MFCC_para7_std, and MFCC_para9_skew) as the most critical predictors of depression across the five machine learning models. MFCC features are highly effective in simulating human auditory processing and align well with human auditory characteristics. They also demonstrate robust recognition capabilities under low signal-to-noise ratio conditions (29). MFCC is commonly viewed as a superior method for identifying differences in vocal emotion characteristics and examining the subtle differences in voice emotions (51). A previous study has found MFCCs are a more stable acoustic feature to reflect the vocal difference between depressed and healthy individuals (45). The density of the spectral feature correlates positively with depression severity, indicating that as depression worsen, the MFCC feature space becomes notably denser (52). Taguchi et al. (43) investigated the differences in MFCC between individuals with and without depression, finding evidence of higher sensitivity and specificity in the second dimension of MFCC, confirming that MFCC may be a distinguishing feature between depression and healthy individuals. One study found that a Gaussian mixture model combined with MFCC could be used to differentiate depression (53). Ozdas et al. (54) reported that MFCC distinguishes depressed individuals from controls with an accuracy rate of 75%. Mobram et al. (55) investigated the accuracy of MFCC features in the depression detection system utilizing the support vector discriminant analysis method was 78%. Altogether, these results suggest that MFCC may serve as objective and valid features for identifying depression from Chinese university students.

## 5 Limitations

Several limitations should be considered. First, this study utilized a convenience sample from a single province in China, which may limit the generalizability of our findings. Future research should incorporate a more diverse, representative sample. Second, we did not adjust for multiple comparisons in this study. Future studies with larger sample sizes will allow for stricter multiple comparison correction methods. Although we applied ten-fold cross-validation, the risk of overfitting remains. Replication of our results in an independent dataset is required.

Third, our classification model was constructed using only one objective measure. Our model employed objective acoustic features to reduce reliance on self-reports, these features may still be indirectly influenced by response biases inherent in the PHQ-9 labels used for model training. Fourth, there may still be unavoidable background noise despite our efforts to create a controlled recording environment. The speech signals collected might contain artifacts related to the acoustic characteristics of the recording space, potentially impacting the extracted acoustic features. Future research should focus on developing more robust feature extraction and modeling techniques that can adapt to different environmental conditions. Lastly, we did not use data augmentation in the study. Some acoustic features may exhibit multicollinearity, but we retained all relevant biomarkers to maximize detection sensitivity in this exploratory phase. This approach preserves clinical information but warrants caution when interpreting individual feature effects. Future studies should aim to enhance the accuracy and reliability of depression recognition by incorporating larger sample sizes, a global profile of acoustic features with uncompressed formats, multi-model clinical assessments, multi-center datasets, data augmentation methodologies or deep learning techniques.

## 6 Conclusions

These findings demonstrate the effectiveness and convenience of utilizing acoustic feature as objective measures to differentiate between control and depression in Chinese university students. The integration of the LDA algorithm with acoustic features can accurately identification of depression, underscoring the significant contribution of MFCC feature in the detection process. This study provides an automated and intelligent acoustic system for large-scale depression screening in Chinese university students.

## Data Availability

The original contributions presented in the study are included in the article/supplementary material, further inquiries can be directed to the corresponding author.
